# The Book World of Medicine and Science

**Published:** 1898-12-24

**Authors:** 


### The Book World of Medicine and Science.

The Schott Treatment of Chronic Heart Diseases. By Richard Greene, F.R.C.P.Ed. Second edition. (London: The Scientific Press, Limited. 1898. Price 6d.)

The second edition of Dr. Greene's little pamphlet will be found useful by those who wish to obtain a general idea of the treatment of heart disease, to undergo which heart sufferers from all parts congregate every summer in the little town of Nauheim. At the present time no doubt the details of the treatment are so generally understood that the *brochure* before us is hardly likely to be of the utility that it was when it first appeared, but those who require the information which is to be found in its pages will find it an interesting little sketch dealing from personal experience with a very important subject.

Shaw's Manual of the Vaccination Law: Containing the Vaccination Acts, 1867, 1871, 1874, and 1898; the Vaccination Order, 1898, and the Instructional Circulars issued by the Local Government Board. By a Barrister-at-Law. Sixth Edition. (London: Shaw and Sons, 1898. Price 5s.)

This is a most useful book, and one which will be found invaluable by those whom it most especially concerns, namely, public vaccinators, vaccination officers, clerks to boards of guardians, and guardians themselves. The book begins with an introduction which, in 37 pages, gives a very fair sketch of the whole subject, treating it historically. Commencing with the discovery of vaccination, it gives shortly the drift of the various Acts of Parliament, and an outline of the inquiry and report by the Royal Commission. Then come the various Acts of Parliament, printed in full, but interspersed throughout with comprehensive notes and cross references which will be found very useful and explanatory; and, lastly, comes the complete text of the Vaccination Order, 1898, with its various Schedules and Forms, the Circular of the Local Government Board as to its operation, and other circulars and notices. Altogether it is a most complete and satisfactory manual.

Gout, Its Pathology and Treatment. By Arthur P. Luff, M.D.Lond., B.Sc., F.R.C.P. (London: Cassell and Company. 1898. Price 5s.)

This book deals with the subject of gout more especially from the standpoint of chemistry, although the account of the clinical aspects of the malady which is given is sufficient to round off the book and make it a fairly complete presentment of the pathology and treatment, as well as of the chemical phenomena of the disease. The first point which the author sets himself to prove is that uric acid in itself is not a poison, and that when it exists in the blood, even in considerable quantity, it produces no evil effect which can be attributed to its presence. It is when it is deposited from the blood as a sodium biurate, which acts passively and physically as a foreign body in the tissues, that it does the mischief and acts as the first cause leading to the gouty manifestations. Then he discusses the question whether the uric acid is a normal constituent of the blood, as it must be if, as some suppose, it is found in the various organs of the body, from which it has to be carried by the blood to its place of exit by way of the kidneys. He concludes that uric acid is not a normal constituent of the blood, but is formed in the kidney and there excreted. His views as to uric acid and gout are expressed in the following passage: "In gout I believe that all the uric acid present in the blood is absorbed from the kidneys, owing to some affection of those organs which interferes with the proper excretion of the uric acid formed in the kidneys. In cases of contracted granular kidney disease, and in cases of plumbism, the uric acid present in the blood is, I believe, derived from the same source, viz., damaged kidneys. In blood diseases and disorders the uric acid present in the blood is probably derived from the nuclein of the leucocytes, and as the kidneys are in a sound condition it is readily excreted by them." This then is the key-note of the book, that gout is not the result of excessive consumption of uric acid, or of bodies of that nature, but is due to deficient excretory power on the part of the kidney. There is, however, just this reservation in regard to the relation between the digestive and metabolic functions and the formation of uric acid, that so far as the latter is derived from glycocine which the liver has failed to convert into urea, the amount of uric acid which the kidney has to form and to secrete may vary much according to the activity of the liver. Needless to say that Dr. Haig's views as to the origin of gout are freely criticised. When we come to questions of treatment, it is not uninteresting to find that some of the drugs which are used by those who believe in their solvent influence are still used, although their solvent influence is denied. Thus, although the treatment of gout by alkalies is decried, citrate of potassium is recommended "for its combined properties of acting as a diuretic, and of diminishing the acidity of the urine." Again, iodide of potassium is given, not, however, as a solvent, but for the reduction of the chronic inflammatory thickening of the fibrous tissue to which the enlargement and tenderness of gouty joints are partly due. In acute or sub-acute cases colchicum is looked upon as most useful. The two things that are not to be used are salicylates and the salts of sodium. Take the book all round, it is likely to be found very useful as a guide to the management of gout, both in its acute and its chronic forms. It is, moreover, a stimulating book to read; its arguments are well put, and, whether we agree with the author or not, it is full of instructive and interesting matter. It is especially useful in that it puts into orderly form and scientific language much that floats all too hazily in the minds of many who are, nevertheless, dogmatic enough in the directions which they give to their patients.

Letts's Medical Diary for the Year 1899. (London: Cassell and Co. Price 2s. 6d. to 5s. 6d., according to size and binding.)

Letts's Medical Diary is so well known that it does not require a word of commendation from us, nevertheless a word may be useful to remind medical men of so useful and well-tried a companion. It is made in two sizes, one for 54 and the other for 108 patients in each week's opening, and is bound in various styles to suit the taste and fancy. In addition to the lists there are various pages ruled for the different purposes required by medical men, such as midwifery and vaccination engagements, nurses' addresses, &c., and some pages of useful information. But this portion is not so extended as to add materially to the bulk of the book.

Eason's Every-Hour Diary, 1899. (Dublin: Eason and Son.)

We would draw attention to the very useful series of diaries issued under the above title. For busy men, such as hospital secretaries, and especially for consultants and dentists, who have numerous engagements, nothing could be more useful, space being provided for each hour, and even for smaller divisions of time. For office use the large size would seem the best, the space provided being ample for the various memoranda which may be required in regard to each engagement. For use in the pocket a small diary is made up for a month. By this means a double page is provided for each day, divided up into hours, and refills can be had for a penny a piece, each serving for a month. There are no advertisements or padding of any sort; just the engagement book which is wanted for the pocket.

